# Recent Development of Plasmonic Resonance-Based Photocatalysis and Photovoltaics for Solar Utilization

**DOI:** 10.3390/molecules21020180

**Published:** 2016-02-02

**Authors:** Wenguang Fan, Michael K. H. Leung

**Affiliations:** Ability R & D Energy Research Centre, School of Energy and Environment, City University of Hong Kong, Kowloon Tong, Hong Kong, China; wg.fan@my.cityu.edu.hk

**Keywords:** visible light absorption, plasmonic photocatalysis, plasmonic photovoltaics, electron oscillation, hybrid nanostructures

## Abstract

Increasing utilization of solar energy is an effective strategy to tackle our energy and energy-related environmental issues. Both solar photocatalysis (PC) and solar photovoltaics (PV) have high potential to develop technologies of many practical applications. Substantial research efforts are devoted to enhancing visible light activation of the photoelectrocatalytic reactions by various modifications of nanostructured semiconductors. This review paper emphasizes the recent advancement in material modifications by means of the promising localized surface plasmonic resonance (LSPR) mechanisms. The principles of LSPR and its effects on the photonic efficiency of PV and PC are discussed here. Many research findings reveal the promise of Au and Ag plasmonic nanoparticles (NPs). Continual investigation for increasing the stability of the plasmonic NPs will be fruitful.

## 1. Introduction

The recent rapid development of solar photovoltaics (PV) has already lowered their cost significantly. However, solar PV is still generally more expensive than the conventional fossil fuel-based energy supply if the environmental cost is excluded. Solar photocatalysis (PC), another significant field of solar energy, has many potential applications to use sunlight directly for functional performance, such as organic pollutant degradation, renewable hydrogen production, self-cleaning exterior building surfaces, *etc.* [1,2,3,4,5]. Titanium dioxide (TiO_2_) is a popular photocatalyst, as it is effective, safe and low-cost. However, the present solar PC reactions are commonly too slow to be practical. The performance is largely limited by the inability to utilize visible light and the fast electron-hole recombination rate [6].

Based on the recent literature, localized surface plasmonic resonance (LSPR) has demonstrated its promise to enhance PV and PC. Past review papers on plasmonic nanoparticles (NPs) have discussed the scientific background and potential applications of LSPR [1,2,3,5,7,8,9,10,11,12,13,14,15,16,17,18,19,20,21,22,23,24,25,26]. Due to the ongoing rapid development of the technologies, in the present review, we especially discuss the recent studies that contribute to plasmonic solar PV and PC using visible light plasmonic NPs.

## 2. Plasmonic Resonance Mechanisms

In highly conductive nanostructures, free electrons are locally confined. When the nanomaterial is irradiated with electromagnetic energy at the plasma frequency, the spatial electron density redistributes and thus creates an electric field; concurrently, a coulombic restoring force of the positively-charged surface nuclei is present and induces collective oscillations of the charges in the particle, similar to an oscillating spring after stretch and release [13]. Such oscillations of electrons and electromagnetic fields are defined as localized surface plasmons. In the state of localized surface plasmonic resonance (LSPR) induced by radiation of a specific LSPR wavelength, the free electrons will oscillate with the maximum amplitude. LSPR is characterized by a build-up of intense, spatially non-homogeneous oscillating electrical fields in the vicinity of the nanostructure [20]. In such a way, the energy of the incident radiation is transferred to the plasmonic particles. The LSPR profile can be tuned by tailoring several parameters, such as nano-size, shape, interparticle distance and the nature of the surrounding medium [2]. The principles of the plasmonic resonance have been discussed in previous review papers [2,13,20,21,22,27,28,29,30].

**Figure 1:**
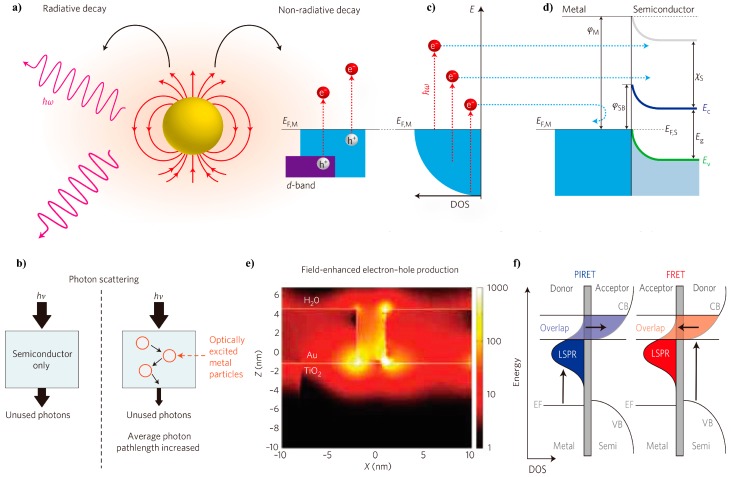
Plasmonic enhancement mechanisms. (**a**) High-energy resonant state decay in two possible forms: re-emission (scattering) of photons or the generation of energetic charge carriers; (**b**) scattering mechanism in which multiple reflections of light among nanocrystals prolong the mean photon path in plasmonic nanostructures and semiconductor composites; (**c**) excitation of electrons from occupied energy levels to a level above the Fermi energy; (**d**) hot electron overcoming the Schottky barrier and injected to the conduction band of the neighboring semiconductor; (**e**) optical simulations using finite-difference time-domain (FDTD) showing SPR-enhanced electric fields around photo-excited Au particles, permeating into a neighboring TiO_2_ structure; electric field intensity normalized by the light source intensity (|E|^2^/|E|_0_^2^) shown by the color bar; (**f**) complementary energy transfer with plasmon-induced resonance energy transfer (PIRET) and Förster resonance energy transfer (FRET) in Au@SiO_2_@Cu_2_O. (**a**,**c**,**d**) Reproduced with permission [22]. Copyright 2014, Macmillan Publishers Limited; (**b**) Reproduced with permission [20]. Copyright 2011, Macmillan Publishers Limited; (**e**) Reproduced with permission [32]. Copyright 2011, American Chemical Society; (**f**) Reproduced with permission [33]. Copyright 2015, Macmillan Publishers Limited.

Upon resonant excitation, the local electromagnetic field in the spatial region around the nanoparticle is intensified. The high-energy resonant state can decay in two possible forms: (1) either through re-emission (scattering) of photons or (2) the generation of energetic charge carriers, as shown in Figure 1a. These charge carriers can induce useful physical or chemical process or relax in the form of heat [21]. The functions of the plasmonic effect on photocatalysis and photovoltaics can be classified into the following three major aspects [31]. They are non-mutually exclusive; in other words, single or multiple mechanisms may contribute to the overall effect.

### 2.1. Optical Enhancement

The LSPR absorption and re-emission of photons are output as a light-scattering function. The form of the light trapping depends on the size of the plasmonic nanoparticles. Smaller nanoparticles primarily localize the electromagnetic field and trigger near-field enhancement (Section 2.3), while bigger nanoparticles mostly scatter the incident photons in the forward and backward directions via an absorption/re-emission form [31]. In this regard, the plasmonic nanostructure functions as nano-mirrors. As illustrated in Figure 1b, the multiple reflections of light among nanocrystals prolong the mean photon path in plasmonic nanostructures and semiconductor composites, resulting in an increased capture rate of incident photons. Unabsorbed incident photons on the first pass through the material can be reflected and scattered by the plasmonic nanoparticles. Such multiple passages of light increase the probability of being captured [2,5,15,18]. This feature is extremely useful when the physical thickness of the light absorption component is limited, such as thin film photovoltaics.

### 2.2. Hot Electron Injection

The electrons are “hot” if their energies are greater than the level of the thermal excitations (Figure 1c). LSPR excitation by visible light can generate hot electrons in nanostructured conductors depending on the size and shape of the nanostructures. The injection of the hot electrons into a semiconductor can improve the solar photovoltaic energy conversion [21,22]. In plasmonic metal/semiconductor hybrid nanostructures, a Schottky barrier exists at the junction interface between the metal and semiconductor. This barrier blocks the electron transfer from either way. However, upon LSPR excitation of the metal nanocrystal, if the hot electrons gain sufficient energy, they can overcome the barrier and inject into the conduction band of the semiconductor (Figure 1d). Actually, the charge injection can occur either from plasmonic metal or semiconductor, depending on the excitation state of the plasmonic metal and the semiconductor. The hot electron injection has been experimentally analyzed in many studies [34,35]. Recently, Ha *et al.* have experimentally demonstrated the two distinct incident energy-dependent charge transfer mechanisms in Au-CdS hybrid nanostructures [35]. Upon the Au LSPR wavelength irradiation, hot electrons were injected from Au to the neighboring CdS; while switched to CdS bandgap irradiation, electron injection from CdS to Au was observed. Both mechanisms showed a preferred response to different incident wavelengths, and they contributed to the generation of reactive catalytic sites. The hot electron injection is similar to the dye-sensitization process in dye-sensitized solar cells. As a result, wide bandgap semiconductors that are originally inactive under visible light gain extra electrons, which can thereafter perform photochemical redox reactions [1,2,7,11,14,20,21,22].

### 2.3. Near-Field Enhancement

The hot electron injection mechanism requires the plasmonic metal and the semiconductor to be in direct contact with each other. However, it was found that even being electrically insulated, the LSPR effect of the metal can still enhance the charge carrier generation of the semiconductor in its vicinity [1,2,17,20,33,36,37,38,39,40]. In plasmonic metal-semiconductor hybrid nanostructures with overlapping LSPR and bandgap energies, incident light in an appropriate spectral region can simultaneously trigger the LSPR excitation of the metal component and bandgap excitation of the semiconductor component. Under such a circumstance, a strong electric field is generated in the vicinity of the metal nanocrystal, whose intensity is several orders of magnitude larger than that of the far-field incident light [2]. This was vividly shown in the optical simulation using the finite-difference time-domain (FDTD) method (Figure 1e) [32]. Because the electron/hole pair generation rate is proportional to the local excitation light, increased generation and separation rates of electron-hole pairs in the part of semiconductor that are subjected to the electric field can be found [1,2,20]. Moreover, introduction of a noble metal component in contact with a semiconductor can often facilitate charge separation upon the generation of electron/hole pairs through photon excitation in the semiconductor, because the Fermi levels of noble metals are typically lower than the conduction band edges of common photocatalytic semiconductors [2].

Another mode of non-radiative near-field enhancement, plasmon-induced resonance energy transfer (PIRET), was proposed based on the findings in experimental and computational simulation results by Wu and co-workers [31,33,36,37,41]. The PIRET was observed for Ag@Cu_2_O, Au@Cu_2_O and Au@SiO_2_@Cu_2_O nanoparticles in photocatalysis applications [33,36,37]. Unlike the SPR-mediated local electromagnetic field enhancement mentioned above, which accelerates the electron-hole pair generation and only uses incident energies above the semiconductor’s bandgap, the PIRET process directly excites electron-hole pairs in the semiconductor non-radiatively through the relaxation of the localized surface plasmon dipole and can occur at energies both above and below the bandgap [33,36,37]. To trigger PIRET, it is required that: (1) the outlying semiconductor needs to be fairly thin to allow reduced recombination losses and an increased absorption within the decay length of the plasmon’s field; (2) a spectral overlap between the semiconductor’s band edge and the LSPR resonance band needs to be maintained [40]; and (3) the relative dephasing times of the plasmon and the semiconductor must be considered, and the back transfer of excited carriers by Förster resonance energy transfer (FRET) on longer timescales must be removed [31,33,36,37]. The difference between PIRET and FRET is illustrated in Figure 1f. Theoretical analysis indicated that PIRET led to the largest enhancement when the plasmon dephasing was close to the semiconductor and the plasmon was overlapped with the band edge [41]. PIRET is particularly useful when charge transfer creates undesirable effects, such as carrier equilibration issues or material degradation [31].

## 3. Plasmonic Photocatalysis

### 3.1. Photochemical Mechanism

**Figure 2:**
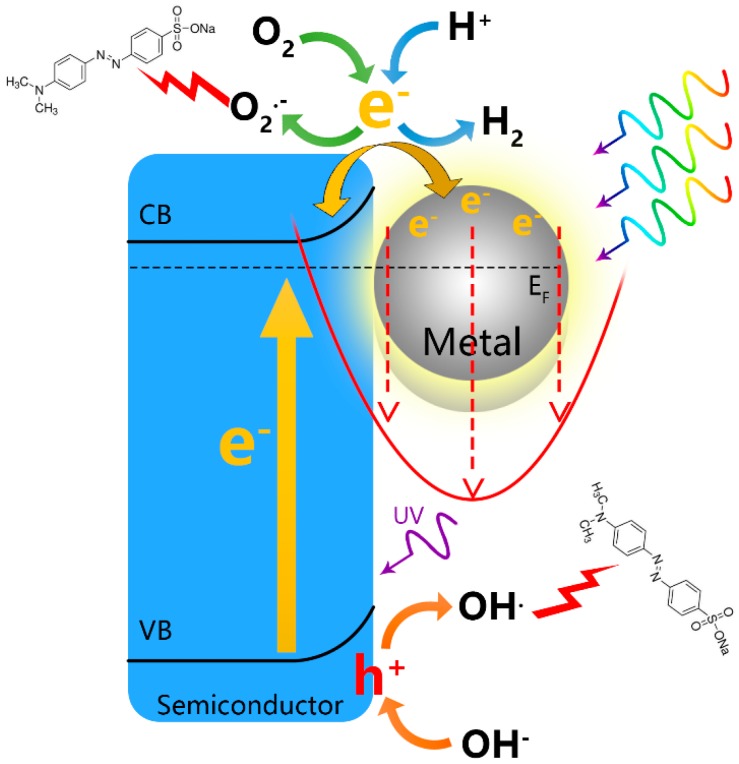
Illustration of plasmonic-mediated photocatalysis. CB, conduction band.

Photocatalysis can be effectively applied to perform photodegradation of environmentally harmful matters, photocatalytic hydrogen evolution and photosynthesis of useful chemicals. The technologies have attracted intensive research interests. The basic mechanisms of nano-semiconductor-based photocatalysis involve photochemical processes of light absorption, electron-hole pair generation and separation and free charge carrier-induced redox reactions, as illustrated in Figure 2. The energy of incident light is thus utilized to drive desirable chemical reactions.

Titanium dioxide is a popular photocatalyst because it is effective, abundant, non-toxic and low cost. However, TiO_2_ suffers from a large bandgap, poor photoabsorption and low photonic efficiency, especially in the low photon energy region. In the pioneering work by Zhao *et al.* [42], the investigators revealed the possibility of visible light utilization by coupling noble metal with TiO_2_. Subsequently, studies of the surface plasmon effect-induced or -enhanced photocatalysis have become a hot research area. In this section, we will review the recent findings and developments in plasmonic photocatalysis.

For plasmonic photocatalysis applications, metal/semiconductor hybrid structures are usually employed. There are some insightful review articles addressing the mechanisms [1,2,22,26]. The charge transfer mechanism also affects the photochemical selectivity of the LSPR-mediated photocatalysis [43]. In the study of SPR-mediated oxidation of *p*-aminothiophenol (PATP), when Au nanoparticles were employed as the catalyst, the LSPR-mediated oxidation of PATP yielded *p*,*p*-dimercaptobenzene (DMAB). When the Au NPs were loaded onto TiO_2_ NPs under LSPR spectra irradiation, no DMAB evolution was detected, as the hot electrons were injected from the Au NPs to the conduction band (CB) of TiO_2_. However, if UV illumination were further introduced, *p*-nitrophenol (PNTP) was formed from PATP in a single step, which was attributed to the electron transfer from UV-excited TiO_2_ to Au NPs. Interestingly, after the UV illumination was removed, PNTP molecules were further reduced to DMAB. These findings indicate that the charge-transfer mechanism may play an important role in LSPR-mediated photocatalysis to manipulate the reaction activity, product formation and selectivity.

### 3.2. Photodegradation of Organic Pollutants

Photodegradation of organic pollutants by semiconductor-based photocatalysts is an economical, clean and effective means for water and air purification. In typical aqueous solution-based photocatalytic reactions, upon photoexcitation, either the photogenerated energetic charge carriers will react with the solvent to form highly reactive radicals (O_2_ + e^−^ → O_2_^−^; OH^−^ + h^+^ → OH), which are able to break the chemical bonds of the organic pollutants, or the charge carriers will be directly transferred to the reactants adsorbed on the surface of the catalysts to induce photochemical transformations. In either way, the organic pollutants are photocatalytically degraded into short-chain molecules or eventually into CO_2_ and H_2_O. The photocatalytic reactions highly depend on the absolute surface area, light absorption properties, energy band level and charge separation potential of the photocatalyst.

The beneficial plasmonic effect on photocatalysis has been well proven. Upon plasmonic excitation, the elevated field increases the generation rate of energetic charge carriers, resulting in a higher probability of redox reactions [19]. Efforts have been made for better morphology control, more suitable energy coupling and novel hybrid structures, in order to achieve higher efficiency and better stability of the catalyst. The performance of recently-developed novel plasmonic nanostructures is summarized in Table 1. Many studies chose organic dyes as the representative pollutant, because the degradation process can be simply monitored through changes in the photo absorption of the reacting solution.

Graphene has also recently received much attention because its electron collecting and shuttling properties are favorable for charge separation to enhance the redox reactions in photocatalysis [44,45]. In addition, a hydrophobic graphene surface creates adsorption sites for pollutant molecules [46]. Therefore, graphene is often chosen as the support for plasmonic hybrid materials [47]. Silver nanoparticles supported by reduced graphene oxide can effectively degrade phenol, bisphenol A and atrazine under UV and visible light irradiation [47]. The study conducted by Cui *et al.* also demonstrated the advantages of the RGO support [48]. In Ag- and Ag_3_PO_4_-decorated RGO, the electron shuttling through RGO separates the oxidation and reduction sites, which is favorable for charge separation. Moreover, the heterostructure also showed better stability than bare Ag_3_PO_4_.

**Table 1:** Plasmonic photocatalytic chemical degradation.

Material/Input Quantity	Pollutant	Pollutant Quantity/Initial Concentration	Irradiation	Performance	Remark	Ref.
Porous TiO_2_-Ag/20 mg	MB	20 mL/50 mg·L^−1^	WL 400 to 700 nm, 95 mW·cm^−2^	98% MB decomposed in 1 h, 2.3-times better than P25	-	[49]
RGO-Ag/22 mg	Phenol, bisphenol A, and atrazine	50 mL/100 mg·L^−1^	UV: ~0.5 mW·cm^−2^ VIS: ~5 mW·cm^−2^	Effective under both UV and visible light	-	[47]
Ag@AgCl/5 mg	MB	50 mL/5 mg·L^−1^	λ > 420 nm	Decomposed in 6 min	Stable in at least 15 cycles	[50]
Ag@AgBr cubic cages/100 mg	MO and RhB	100 mL/10 mg·L^−1^	Xe lamp with 400 nm UV cutoff 72 mW·cm^−2^	MO within 80 s and RhB within 160 s under visible light	Stable in at least 7 cycles	[51]
Ag@C_3_N_4_ core-shell nanocomposite/25 mg	MB	50 mL/0.01 mM	Xe lamp with 420 nm UV cutoff 38 mW·cm^−2^	>85% decomposed in 1 h	-	[52]
g-C_3_N_4_/Ag/TiO_2_ microspheres/30 mg	MO and Phenol	MO: 30 mL/13.5 mg·L^−1^ Phenol: 30 mL/16.6 mg·L^−1^	300-W Xe lamp with 420 nm UV cutoff	94% MO degraded in 6 h under visible light; phenol degradation showed similar trend	MO degradation decreased from 94.0% to 86.5% after four cycling runs	[53]
Ag/AgVO_3_/50 mg	Basic fuchsin dye	50 mL/20 mg·L^−1^	500-W Xe lamp with 420 nm UV cutoff	93.6% of BF decomposed within 90 min k = 0.0286 min^−1^	-	[54]
AgIn(MoO_4_)_2_nanosheets grafted Ag/AgBr/100 mg	Tetracycline	100 mL/10 g·L^−1^	500-W Xe lamp with 400 nm UV cutoff	~60% degraded in 20 min k = 0.038 min^−1^	-	[55]
RGO-supported Ag and Ag_3_PO_4_/50 mg	RhB and phenol	50 mL/20 ppm	250-W tungsten halogen lamp with 400 nm UV cutoff	90.5% RhB decomposed in 30 min k = 0.1411 min^−1^	Stability better than bare Ag_3_PO_4_	[48]
Fe(III)/Ag-Ag_3_PO_4_/100 mg	MO	10 mL/20 mg·L^−1^	Xe lamp with 400 nm UV cutoff 40 mW·cm^−2^	k = 0.038 min^−1^	-	[56]
Au-CdS spherical nanoparticles/30 mg	RhB	60 mL/10 mg·L^−1^	350-W Xe lamp	78.9% RhB decomposed in 10 min	-	[57]
Dumbbell-like Au-Bi_2_S_3_-CdS core-shell nanorods/10 mg	RhB	30 mL/10^−5^ M	300-W Xe lamp	64.2% RhB decomposed in 10 min, 2-times faster than P25	-	[58]
Au@ZnO/5 mg	MO	50 mL/62 mM	150-W Xe lamp with 390 nm UV cutoff	64.5% decomposed in 60 min	-	[59]
Au-loaded N:TiO_2_/1 g·L^−1^	Formic acid	1086 μmol·L^−1^	UV: 4.2 mW·cm^−2^ at 365 nm VIS: photon flux = 85 × 10^−3^ μmol·s^−1^	Under UV: degraded in 30 min, 2-times faster than P25 Under visible light: 30% degraded in 6 h, while P25 showed no activity	-	[60]
Au/TiO_2_ 3D hollow nanospheres/30 mg	Isopropanol	Gaseous, amount unspecified	300-W Xe lamp with 420 nm UV cutoff	7.4 μmol of CO_2_ generated under 10 h visible light, 6.1-times higher than Au/P25	-	[61]

### 3.3. Photocatalytic H*_2_* Generation by Water Splitting

The essential reaction of hydrogen generation from water splitting is the proton reduction (at pH = 7):

(1) $$2H^{+}+2e^{-}\to H_{2},{\text{E}}^{0}=-0.41\;\text{V}\;vs.\;\text{NHE}$$

The main purpose of water splitting is to convert the solar energy into hydrogen and store it as a fuel. Therefore, the photocatalysts’ ability of light absorption and conversion in the solar spectrum is the first consideration. Efficient generation of hydrogen relies on sufficient free electron energy (more negative than E^0^) and the separation of the photogenerated charge carriers. Recombination is a limiting factor of the energy conversion efficiency. Moreover, filling of the photogenerated holes is also crucial, not only because it can promote the electron injection, but also it prevents the catalyst from photocorrosion.

Additional plasmonic component can improve the water splitting effect. The three major functions of plasmonic enhancement, *i.e.*, (1) the photon scattering; (2) hot electron transfer and (3) near field enhancement, are effective if the nanostructure is assembled in a proper way. The detailed mechanisms and previous studies have been reviewed elsewhere [1,13,14,62]. Here, we mainly review the most recent findings and novel designs in plasmonic water spitting.

Plasmonic materials can increase the generation rate of energetic charge carriers in photocatalytic water splitting. However, since the charge carriers could rapidly recombine without any productive reactions, effective charge separation has been identified to be the most important strategy to improve the performance of the photo-chemical conversion. The charge separation properties highly depend on the interfacial design of the nanostructures. For the Au-TiO_2_ combination, as the support of Au NPs, anatase/rutile composites (P25 and Hom) were found to exhibit the highest H_2_ evolution rates under both UV-VIS and visible light [63]. The phenomenon was attributed to an improved charge separation due to the electron transfer across the anatase/rutile interface in both directions, depending on the excitation wavelength. In contrast, pure anatase and anatase/brookite-supported Au NPs showed no H_2_ evolution under visible light. It was found via *in situ* EPR spectroscopy that an intrinsic accumulation of Ti^3+^ in the lattice of these supports may increase the Schottky barrier at the Au/TiO_2_ interface, which thereby hindered LSPR-promoted electron transfer from Au to TiO_2_ [63].

The Schottky barrier at the interface of metal-semiconductor junctions plays a crucial role in charge separation [34,63,64,65]. It was found to be able to prolong hot electron lifetimes in Au NP-decorated TiO_2_ nanorod arrays by suppressing recombination of the hot electrons injected to the TiO_2_ CB [64]. The open-circuit decay photoelectrochemical techniques were used to analyze the mechanics of LSPR-mediated electron transfer within the Au/TiO_2_ heterostructures under visible light irradiation (λ > 515 nm), compared to the electron lifetimes in TiO_2_ via UV excitation. It was found that the plasmon-mediated hot carriers displaying excited-state lifetimes are two orders of magnitude longer than those with bare TiO_2_ [64]. As a result, good performance of PEC water splitting was achieved at a rate of 0.9 ± 0.1 μmol H_2_/cm^2^·h, in the presence of methanol as a sacrificial reagent [64].

**Figure 3:**
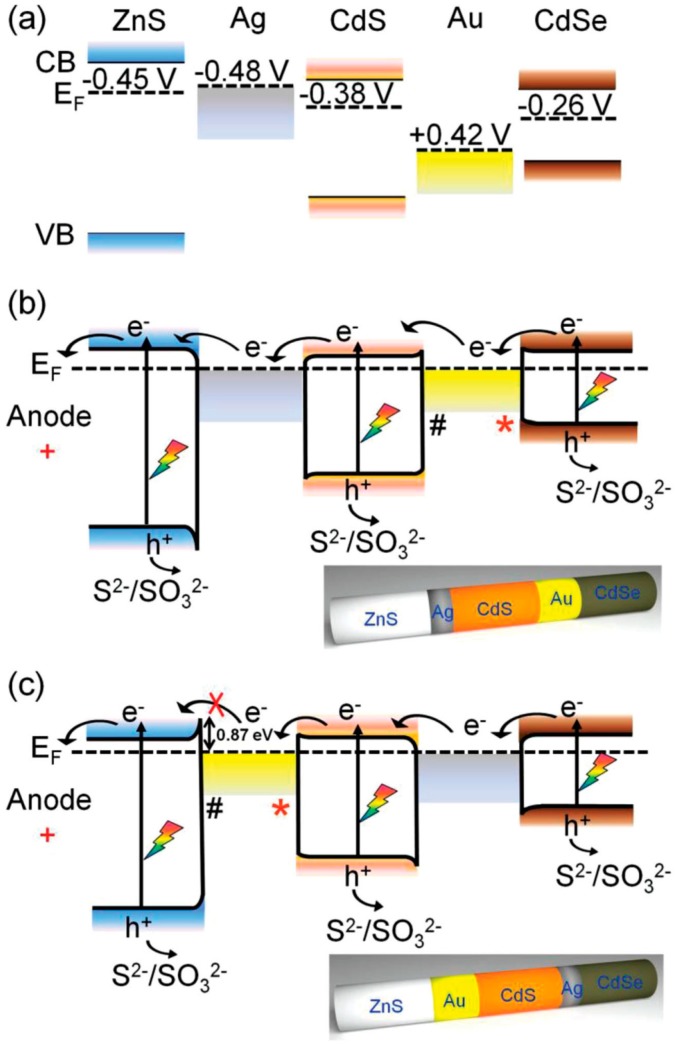
(**a**) Energy levels of ZnS, CdSe, CdS, Au and Ag (*vs.* NHE); illustration of the interfacial PICT processes in (**b**) ZnSAg-CdS-Au-CdSe nano-bamboo and (**c**) ZnS-Au-CdS-Ag-CdSe nano-bamboo after heterostructuring. *: forward-bias Schottky junction; #: reverse-bias Schottky junction. Reproduced with permission [65]. Copyright 2015, Wiley.

Although the Schottky barrier has been proven to facilitate the charge separation, the role of it is not always positive [13,20,34,65]. For complicated designs of nanostructures, manipulating and tuning the charge carrier transport within the heterostructures is the key for achieving good performance: a good design can facilitate the interfacial charge separation and inhibit the charge recombination, resulting in higher photocatalytic efficiency. On the other hand, inappropriate assembly of various components would lead to serious recombination losses, as an unsuitable interfacial energy band structure would result in undesired energy barriers at the heterojunction and impede the charge transportation [6,65]. This was demonstrated in a recent study, as shown in Figure 3, in which a nano-bamboo array architecture comprising five components (ZnS, Ag, CdS, Au and CdSe) was employed for photoelectrochemical water splitting [65]. Two different component alignments (Figure 3b,c) were studied. While having the similar spectral absorption and geometry, the ZnS-Ag-CdS-Au-CdSe setting (Figure 3b) exhibited much better performance (8.57 mA·cm^−2^ at 0 V *vs.* Ag/AgCl) than the ZnS-Au-CdSe-Ag-CdS (Figure 3c) counterpart (2.22 mA·cm^−2^) [65]. The drastic difference in PEC performance was attributed to the difference in the configurations of plasmonic-metal/semiconductor interfaces [65]. As shown in Figure 3a, since the Fermi level of Au is lower than that of both CdS and CdSe, the interfacial Schottky barriers are generated at both CdS/Au and Au/CdSe interfaces [65,66]. When the photoanode was implemented in the PEC cell and a positive bias (relative to the counter electrode) is applied, forward bias (* in Figure 3) and reverse bias (# in Figure 3) Schottky junctions are generated at the Au/CdSe and CdS/Au interfaces, respectively [65,67]. The forward-biased Schottky junction enables easy flow of electrons across the Au/CdSe interface, while the reverse-biased Schottky junction at the CdS/Au interface might impede the electron transportation between the two components [67]. According to the electron pathways illustrated in Figure 3, the high-performance ZnS-Ag-CdS-Au-CdSe benefited from the suitable interfacial band arrangements, which allowed easy electron transport and effective charge separation, while the ZnS-Au-CdSe-Ag-CdS setting suffered from a reverse bias Schottky junction at the ZnS/Au interface, which led to undesired charge recombination and, thus, low-efficiency photoconversion [65].

A similar conclusion was also drawn from other studies of multi-component nanomaterials: Au@CdS/SrTiO_3_ [34] and Au-incorporated SrTiO_3_-AgNbO_3_-Pb0.86La0.14TiO_3_ perovskite oxide combinations [68]. In the study by Yu *et al.* [34], the electron behavior was monitored by a set of control experiments. The Schottky barrier between Au and SrTiO_3_ was again proven to be beneficial for the separation of hot electrons and holes. It is noteworthy that in one of the control experiments, some Pt NPs were further decorated onto to the surface of the SrTiO_3_. The H_2_ production rate was further improved by 5.62-fold (from 29.1 μmol·h^−1^ to 163.6 μmol·h^−1^). The improvement was attributed to the electron sinking effect of the Pt NPs. The proposed mechanism is that upon LSPR excitation of the Au cores, a fraction of the transiently-produced hot electrons overcome the Schottky barrier at the Au/SrTiO_3_ interface and are injected into the CB of SrTiO_3_. The electrons then diffuse to the surface of the SrTiO_3_ and are subsequently entrapped by the Pt NPs, which served as active sites for H_2_ evolution. Meanwhile, the residual holes in the Au cores are filled by the electrons injected from the simultaneously photoexcited CdS shells. Finally, the holes in the CdS shells are quenched by sacrificial ions in the solution. In the study by Shieh *et al.* [68], it was reported that the outcome of the plasmonic enhancement was strongly dependent on the location of the plasmonic Au NPs. Good design can increase the photocurrent through the light scattering effect and the near-field effect; contrarily, improper positioning of the Au NPs would render the NPs as recombination centers, which harms the performance [68].

**Figure 4:**
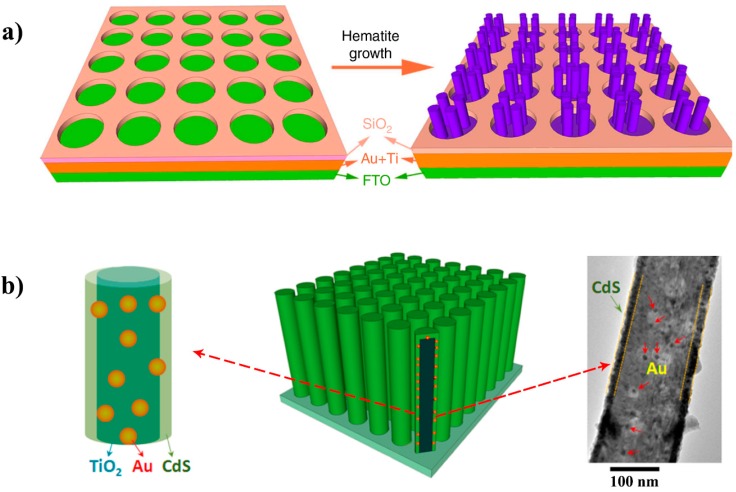
Schematic diagram for the architecture design of (**a**) the growth of the hematite nanorod array on the Au nanohole array and (**b**) CdS-Au-TiO_2_ sandwich nanorod array. (**a**) Reproduced with permission [69]. Copyright 2014, American Chemical Society; (**b**) Reproduced with permission [70]. Copyright 2013, Macmillan Publishers Limited.

Besides the size and shape of the plasmonic nanoparticles, the architecture design of the metal-semiconductor heterojunction plays an important role in determining the features and performance of the hybrid materials. Wu and co-workers designed a hematite nanorod array incorporated into a plasmonic gold nanohole array pattern for photoelectrochemical water splitting, as illustrated in Figure 4a [69]. The ordered plasmonic Au nanohole array pattern was firstly fabricated using nanosphere lithography on a fluorine-doped tin oxide (FTO) substrate (thickness: 85 nm; diameter: 350 nm; and center-to-center separation: 490 nm) for surface plasmonic polariton (SPP) and LSPR modes at the wavelengths below and above the band gap of hematite [69]. Then, the hematite nanorods were grown on the Au nanohole array pattern. The unique design allowed the following three mechanisms occurring in tandem: (1) above the absorption band edge of hematite, the SPP launched a guided wave mode inside the nanorods, making the nanorods function as miniature optic fibers, enhancing the light absorption; (2) below the band edge, since a spectral overlap between the hematite’s band edge and the LSPR resonance band existed, the photo energy collection at the energies below the band edge was enhanced through the PIRET mechanism; (3) the intense local plasmonic field suppressed the charge recombination in the hematite nanorods [69]. As a result, the photocurrent at a bias of 0.23 V *vs.* Ag/AgCl was increased approximately by a factor of 10 compared to the pure hematite nanorod array under simulated solar radiation [69]. Later, the same group reported another system with an innovative structure design, the CdS-Au-TiO_2_ sandwich nanorod array, as shown in Figure 4b [70]. Based on the transient absorption spectroscopy analysis, it was demonstrated that the addition of Au nanoparticles increased the charge-transfer lifetime, reduced the trap state Auger rate, suppressed the long time scale back transfer and partially compensated the negative effects of the surface trap states [70]. The Au nanoparticles was found to play a dual role in enhancing the solar-to-hydrogen conversion efficiency: (1) functioning as a sensitizer to extend the photoconversion spectrum to 725 nm and (2) functioning as electron relay to facilitate charge transfer between the CdS and TiO_2_ [70]. As a result, a photocurrent density of 4.07 mA/cm^2^ at 0 V *vs.* Ag/AgCl was obtained under full solar spectrum irradiation, and the maximum solar-to-chemical energy conversion efficiency reached 2.8% [70].

Another way of improving the charge separation is employing graphene as the support for the functional catalysts [71,72,73,74]. Graphene-supported hybrid nanostructures, including Ag-TiO_2_ [73], Au-TiO_2_ [71], Au-N:La_2_Ti_2_O_7_ [75] and CdSe/CdS-Au [72], were applied in photocatalytic hydrogen generation. The results of all of these studies suggest that the introduction of graphene support can efficiently improve the performance. The improvement was attributed to the increased reactive surface area, as well as the superior charge separation brought about by the graphene support. The unique electron collecting and shuttling properties of graphene drive the hot electrons to reactive sites, thus suppressing the recombination.

### 3.4. Photocatalytic CO*_2_* Reduction

Photocatalytic CO_2_ reduction is also a solar-to-fuel conversion process. It is particularly attractive due to its simultaneous reduction in greenhouse gas emission. Carbon dioxide molecules are stable and chemically inert. The single-electron reduction of CO_2_ to an anion radical CO_2_^−^ has a strongly negative electrochemical potential of −1.9 V (*vs.* NHE at neutral pH), which virtually no semiconductor can overcome [76]. Therefore, the photocatalytic CO_2_ reduction is usually achieved by multiple electron transfer with the assistance of a corresponding number of protons. Equations (2) to (6) present the electrochemical CO_2_ reduction potentials to formic acid, carbon monoxide, formaldehyde, methanol and methane, respectively (*vs.* NHE at pH = 7) [76].

CO_2_ + 2H^+^ + 2e^−^ → HCOOH E^0^ = −0.61 V (2)

CO_2_ + 2H^+^ + 2e^−^ → CO + H_2_O E^0^ = −0.53 V (3)

CO_2_ + 4H^+^ + 4e^−^ → HCHO + H_2_O E^0^ = −0.48 V (4)

CO_2_ + 6H^+^ + 6e^−^ → CH_3_OH + H_2_O E^0^ = −0.38 V (5)

CO_2_ + 8H^+^ + 8e^−^ → CH_4_ + 2H_2_O E^0^ = −0.24 V (6)

Unlike photocatalytic water splitting, which normally takes place at a solid-liquid interface and requires no additional oxidizing or reducing agents, photocatalytic CO_2_ reduction usually takes place at either a solid-liquid or a solid-gas interface, and an electron donor/reducing agent that acts as the hydrogen source is required O. Water is usually employed to act as the reducing agent [77]. However, the concurrent generation of hydrogen as described by Equation (1) competes with carbon species for the photogenerated electrons, which should be suppressed. Moreover, since photocatalytic CO_2_ reduction is a complex process in general and involves multiple intermediate radicals and redox reactions, the selectivity of the final product is important [76,77]. All of the above concerns pose an extra challenge to develop the photocatalytic CO_2_ reduction for large-scale application.

LSPR-mediated photocatalytic CO_2_ reduction has been proven favorable, as discussed in some previous insightful reviews [2,14,76]. Recently, several studies have reported the use of plasmonic Au or Ag nanoparticle-decorated TiO_2_ materials for photocatalytic CO_2_ reduction to methane under UV or visible light [77,78,79,80]. It was generally found that the plasmonic nanoparticles could promote the transformation from CO_2_ to methane, especially in the visible light spectrum. The loading ratio between plasmonic metal and TiO_2_ could affect the selectivity of the products. Lower loadings of Au resulted in a preference of the hydrocarbon production, especially CH_4_ [77]. Besides methane, methanol could also be obtained with the catalysts of Ag/TiO_2_ nanocomposites [81] and Cu-modified TiO_2_ nanoflower films [82]. Liu *et al*. reported that a composite of 2.5% Ag/TiO_2_ exhibited the best activity, and the methanol yield was 135.1 μmol g-catal.^−1^·h^−1^ under UV and visible light irradiation, which was 9.4-times higher than that of pure TiO_2_ [81]. Later, the same group reported CO_2_-to-methane conversion using Cu-modified TiO_2_ nanoflower films, and the performance was 6.0-times higher than a pure TiO_2_ film under UV and visible light illumination [82]. Tahir *et al.* used hydrogen gas as the reducing agent instead of water, and reduced CO_2_ to CO, CH_4_ and methanol with Au nanoparticle-modified TiO_2_ nanowires as the catalyst under visible light irradiation [83]. The highest activity was obtained at 0.5 wt % Au loading [83]. Kawamura *et al.* also used H_2_ as the reducing agent and carried out CO_2_ reduction over assemblies of Au/Zn_3_Ga(OH)_8_]_2_CO_3_·mH_2_O and Ag/Zn_3_Ga(OH)_8_]_2_CO_3_·mH_2_O as photocatalysts exposed under both UV and visible light [84]. The CO_2_ photoreduction rate using Ag/Zn_3_Ga|CO_3_-IE catalyst was higher by a factor of 1.69 in comparison with that of Zn_3_Ga|CO_3_ [84]. Moreover, the Au/Zn_3_Ga|CO_3_-reconst was CO selective (87 mol %) and showed the best catalytic performance (231 nmol g-catal.^−1^·h^−1^) [84].

### 3.5. LSPR-Mediated Photosynthesis

For the synthesis of useful chemicals, especially of those having complicated molecules, the introduction of photocatalytic mechanism would shift the apparent thermodynamic equilibrium to the favorable side of the redox reactions at low temperature to avoid thermally-induced side reactions [85]. In the early stage of development, when semiconductors of a large bandgap were employed as the photocatalyst, ultraviolet (UV) light was commonly required to activate the reactions. There are two problems arising from the UV light activation: (1) limited yield due to only a small fraction of solar radiation being useful and (2) poor selectivity for the desired product due to the reliance on free radical intermediates in UV-induced chemical reactions [85]. Plasmonic photocatalysts possess unique properties that can (1) enable difficult reactions to take place under mild conditions [86,87]; (2) exhibit excellent selectivity of products [43,86,87] and (3) improve the performance of existing catalysts [88].

Guo *et al.* have investigated graphene-supported copper nanoparticles for coupling reactions of aromatic nitro compounds to the corresponding azoxy and azo compounds under visible light irradiation [86]. The surface plasmon-mediated energy transfer from the incident light to the electrons of the copper nanoparticles facilitates the breaking of the N-O bonds in the aromatic nitro compounds. When irradiated with natural sunlight (mean light intensity of 0.044 W·cm^−2^) at about 35 °C, 70% of the nitrobenzene is converted, where 57% of the product is azobenzene. This means the catalyzed coupling reaction can proceed under moderate conditions with the assistance of natural solar energy. It was also found that at 60 °C, the coupling of nitrobenzene produces azoxybenzene with a yield of 90%, while at 90 °C, the product was azobenzene with a yield of 96%. The wavelength of the incident photons can also affect the outcome. Therefore, the product can be selected simply by tuning the reaction temperature or the irradiation wavelength.

In another sample, bi-modal Au nanoparticles (2 nm and 9 nm) were loaded on rutile titanium oxide for one-step photosynthesis of azobenzenes from nitrobenzenes under visible light at room temperature [87]. A yield greater than 95% and a selectivity greater than 99% were achieved. Control experiments showed that uni-modal hybrid structures were rather inactive compared to the bi-modal counterpart. This phenomenon indicates that upon the LSPR excitation, electrons are transported from smaller Au NPs to larger Au NPs through the CB of TiO_2_. As electrons accumulate in the larger NPs, they function as reduction sites where nitrobenzene undergoes eight-electron reduction to azobenzene via azoxybenzene by their electron pool effect. On the other hand, the holes left in the smaller NPs oxidize the 2-propanol solvent to acetone.

For the formation of carbon-carbon bonds, palladium-based catalysts are widely used in the cross-coupling reactions. Conventionally, many of the catalytic reactions are thermally driven to achieve viable efficiency. The problems include an energy-intensive process, the possible formation of unwanted side-product and impaired stability and reusability of the catalysts [88]. Some groups have found that with the introduction of Au, either via alloying with Pd [88,89] or in the form of bimetallic nanostructures [90,91], the cross-coupling reactions were enabled to occur under milder conditions with photoactivation. In a recent study, five different cross-coupling reactions, namely the Sonogashira, Stille, Hiyama and Ullmann C−C couplings and the Buchwald–Hartwig amination (C−N cross-coupling) were investigated to examine the applicability of Au-Pd alloy NP photocatalysts under visible light irradiation [88]. It was demonstrated that the photocatalytic effect can significantly enhance the intrinsic catalytic activity of Pd at a lower temperature for these cross-coupling reactions. The alloy NPs showed the ability to concentrate the incident photon to a very small volume and to transfer this energy to the surface of Pd sites, where the reactant molecules were adsorbed and, thus, ignite the cross-coupling reactions. As a characteristic of the plasmonic effect, the intensity and wavelength of the light irradiation also play a key role in the catalytic performance.

## 4. Plasmonic Photovoltaics

### 4.1. Photoelectrochemical Mechanisms

Solar cell technologies have undergone several generations of development, from the traditional silicon solar cells to the most recent perovskite solar cells. The fundamental working principles of photovoltaics cover the following processes:

1. *Light absorption*: to optically confine the solar radiation into the active special region of the device;
2. *Energy transfer*: energy of the incident photons is transferred to free charge carriers through photoexcitation;
3. *Charge separation and transportation*: the free charge carriers have to be driven into different directions to form an electric current and to avoid recombination;
4. *Charge collection and restoration*: the charge carriers are collected by the electrodes after the circulation and restored to maintain the stability of the system.

**Figure 5:**
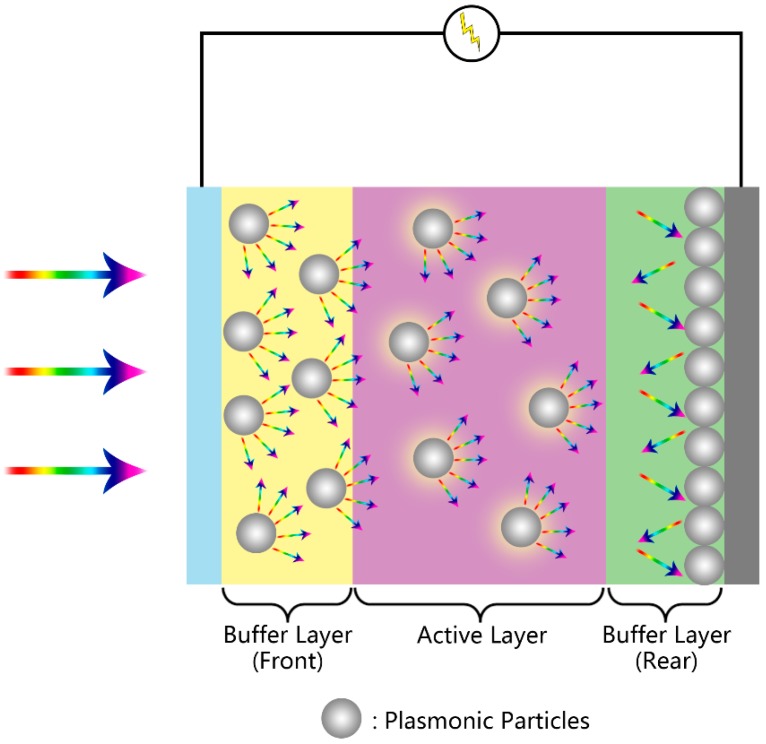
Mechanisms of plasmonic enhanced Si/polymer photovoltaics.

For the advancement of PV technologies, new initiatives to improve one or multiple processes are required. In this context, plasmonics has gained increasing attention as one of the best solutions in many aspects. Some insightful reviews on the fundamentals and applications of plasmonic photovoltaic technologies are available in the literature [8,9,10,17,22]. The plasmonic PV mechanisms are illustrated in Figure 5. In this section, we discuss the recent developments in plasmonic photovoltaics.

### 4.2. Silicon Solar Cell

Light absorption is the first step of the solar energy conversion. It defines the amount of energy that can be potentially converted by the device. In conventional Si solar cells, light trapping is typically achieved using a pyramidal surface texture that causes scattering of light into the solar cell over a large angular range, thereby increasing the effective path length in the cell [17]. Recently, Liu *et al.* have proposed a simple chemical etching method to incorporate nanopits into the pyramidal structures, with Ag nanoparticles embedded in the nanopits [92]. An enhanced light trapping was achieved through the hybrid nanostructure by the medium effect of the nanopits and the LSPR effect of the embedded Ag NPs. The EQE was increased to 95% and J_SC_ by 17% for the cells without SiNx anti-reflection coating [92].

For the amorphous silicon thin film-based solar cells, light absorption issues have even more impact on the overall cell performance; because the amorphous silicon has a very big reflection index and exhibits poor near bandgap absorption. Gusak *et al.* has reported underlying high aspect ratio plasmonic nanocones beneath the ultra-thin amorphous silicon layer to achieve enhanced light absorption [93]. Wang *et al.* deposited a mixture of Ag and Au nanoparticles on top of the Si layer and increased the PCE by 5% [94]. Another route is to incorporate a nanostructured plasmonic back reflection layer in the rear surface of the device. Self-assembled Ag NPs as the back reflection layer increased the PCE from 4.33% to 5.42% [95], while bimetallic Au/Ag multispiked NPs boosted the PCE from 6.72% to 7.7% in an c-Si/polymer solar cell [96].

### 4.3. Organic Solar Cell

In organic polymer cells, the main bottleneck in achieving high power conversion efficiencies is the tradeoff among light absorption and charge carrier generation, transportation and collection [2,9,15]. The physical thickness of active layers (ALs) has to be controlled at a sub-optimal level due to the poor charge carrier mobility and small exciton diffusion length of most molecular and polymeric materials being used in such devices. However, such a setting leads to poor light absorption, resulting in low power conversion efficiencies [58]. Realizing optically thick, yet physically thin functional layers has become a major challenge in PV research. Due to the magnificent optical properties of plasmonic structures, the incorporation of plasmonic elements into thin film photovoltaic devices has shown promising outcomes [5,24]. Recent findings are summarized in Table 2.

For the incorporated plasmonic nanostructures, it has been generally accepted and demonstrated that the optical and plasmonic profiles would be significantly affected by the metal, shape, size, *etc.* [97,98,99]. For example, where Au@SiO_2_ nanospheres and nanorods were applied under the same situation, different light absorption spectra were observed, as well as the power conversion performance. Nanorods significantly outperformed nanospheres [99]. The reason was attributed to more light scattering and a higher quality factor for LSPR triggered by nanorods. Another frequently employed morphology is nanoprisms [98,100,101,102]. Nanoprisms exhibit relatively larger scattering to absorption ratios than nanospheres and possess much stronger optical antenna effects, with larger local field enhancements [98,102]. In the structural engineering view, the flat shape of prisms allows for a larger optical cross-section with a small thickness in the plane of the device, which maximizes optical interactions while minimizing the risk for shorting [98]. However, nanoprisms with sharp corners have high structural energy and, thus, are easy to lose shape or be decomposed [102,103]. It was found that Au or Au-capped nanostructures exhibited better photo and chemical stability than Ag nanostructures [103]. Enhanced stability was achieved by coating Au at the edge of Ag nanoprisms [100] or encapsulate the Ag nanoprisms in TiO_2_ or SiO_2_ shells [101,102].

Besides the morphologies, the positioning of the plasmonic elements also raised different arguments, especially for polymer solar cell. Due to the short decay lengths of LSPR modes, the absorber needs to be within a short distance of the metal nanostructure, such that there is substantial overlap with the plasmonic near-field, in order to fully benefit from the near-field enhanced absorption [104]. For such a concern, placing the plasmonic nanomaterials right in the active layer would be the most ideal solution, as significant enhancements have been reported in many studies [99,101,105,106,107,108]. For example, incorporating Au NP-decorated nitrogen (N)- or boron (B)-doped carbon nanotubes into the bulk heterojunction layer of the PTB7:PC_70_BM polymer solar cell was found to bring synergistic enhancements of light absorption, charge generation, dissociation and transport properties [106]. The enhanced light absorption and charge generation was attributed to the LSPR effect of the Au NPs [106]. The PCE was increased to as much as 9.98% from that 8.12% of the reference device [106]. However, it was found that incorporation of metal particles into the active layer can be problematic because (1) the particles can act as recombination centers and (2) the particles and their associated processing additives can also influence the sensitive morphology of a polymer solar cell bulk heterojunction [15,98,101,107]. Extra means have to be taken to avoid such problems. It was found that coating the metal NPs with a SiO_2_ or TiO_2_ layer can effectively suppress the recombination at the metal NPs [99,101,107]. Another alternative approach is to incorporate the plasmonic structures into the electron- and/or hole-transport interfacial layer(s) [98,105,109,110,111,112]. The general route of this kind of incorporation is to blend pre-synthesized plasmonic particles with interfacial polymer solutions (for example: PEDOT:PSS) and cast the mixture in the same way. Yao *et al.* demonstrated that this strategy was applicable to a number of combinations of organic solar cells [98]. Moreover, it was shown that incorporating plasmonic structures into both the hole transport layer (HTL, front) and electron transport layer (ETL, rear) can further enhance the PCE, since the unused photons can be reflected multiple times within the cell; thus, the mean optical path was increased [98].

**Table 2:** Characteristics of plasmonic organic solar cells.

Plasmonic Material	Position *	Enhancement in PCE (η)	Remark	Ref.
Patterned Ag/PVP Electrospun Nanofibers	Between ITO and HTL	3.53% → 4.19%	-	[97]
Au NP-GO	As the HTL	3.26% → 3.78% (P3HT:PCBM), 4.02% → 5.05% (P3HT:ICBA)	P3HT:ICBA AL showed increased Voc	[109]
Bone-like Au NPs with other Au nanostructures	Between HTL and anode	3.27% → 4.06%	Enhanced absorption at 300 to 1000 nm	[113]
Hemispherical Ag nanostructure	Between ETL and cathode	5.75% → 7.18%	Exhibited long-term stability and an extended lifetime	[114]
Ag nanoplates	In HTL	3.8% → 4.2% (P3HT:PCBM), 6.1% → 6.4% (PTB7/PC_71_BM)		[115]
Au NPs	In HTL	8.66% → 9.06%	-	[110]
Al@PPh_3_ NPs	In HTL	5.26% → 6.29%	-	[111]
Ag NPs	In ETL	2.0% → 3.2%	Enhancement was only observed when the Ag NPs were fully embedded into the ETL	[112]
Silver plasmonic nanoprisms	In HTL/ETL/both	7.7% → 9.0%	Highest PCE achieved by dual-type, applicable to multiple cell types	[98]
Ag NPs	In HTL and AL	7.45% → 9.04%	Different NP sizes in HTL and AL at optimization	[105]
Au-CNT nanohybrids	In AL (and HTL)	8.12% → 9.75%	Highest PCE was achieved with additional Au NPs incorporated in HTL	[106]
Au@SiO_2_ nanospheres and nanorods	In AL	6.5% → 8.2%	Claimed no adverse impact on the device stability with NRs in AL	[99]
Ag@SiO_2_ NPs	In AL	3.44% → 3.96%	Bare Ag NPs (W/O SiO_2_ coating) decreased PCE	[107]
Ag@ (TiO_2_ or SiO_2_) nanoprisms	In AL	3.1% → 4.03%	Outlying oxides suppressed recombination	[101]
Au and Al NPs	In AL	5.33% → 6.12%	-	[108]
Ag nanoprisms with Au-coated edges	Beneath AL	NA	Claimed high stability of plasmonic structures and increased charge carrier generation	[100]
Graphene nanosheets/AgNPs	As Interfacial Layer Between ETL & AL	3.38% → 4.04%	-	[116]
Au NPs	Between ETL & Cathode	2.3% → 3.6%	-	[117]

* Note: HTL, ETL and AL represent hole transport layer, electron transport layer and active layer, respectively.

### 4.4. Dye/Quantum Dot-Sensitized Solar Cell

**Figure 6:**
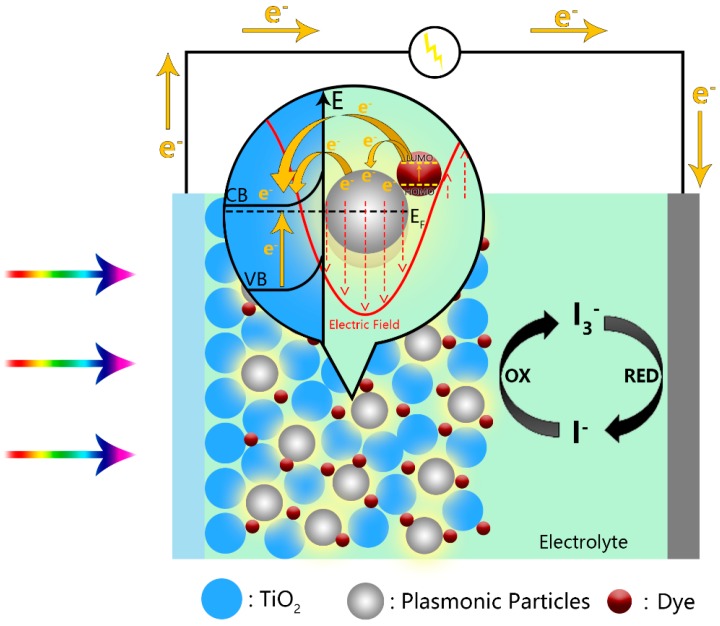
Illustration of plasmonic dye-sensitized solar cell.

Sensitized solar cells, mainly referring to dye or quantum dot-sensitized solar cells, are based on the sensitization of mesoporous, nanocrystalline, semiconductor films (typically a network of TiO_2_ nanoparticles), towards the solar spectrum through the adsorbed molecular dyes or semiconductor quantum dots. The schematics is shown in Figure 6. The solar-to-electricity conversion performance largely depends on the photoelectrode’s ability to harvest incident light and separate the photo-generated charge carriers. The mechanisms of plasmonic enhancement discussed above can be well extended to sensitized solar cells. In fact, the introduction of plasmonic elements has been an intensively studied strategy in past and recent research.

The most employed incorporation method is to blend as-prepared plasmonic nanostructures into the precursor paste of the mesoporous TiO_2_ and cast the mixture onto the substrates [102,118,119,120,121,122,123,124,125,126,127,128,129]. After heat treatment, the plasmonic particles can be dispersed into the mesoporous layer and function as light harvesting and charge separation sites. Increased PCE comparing to conventional bare TiO_2_ mesoporous substrates were observed in these studies. Besides precursor blending, several different incorporation methods were also applied. Adhyaksa *et al.* used an electrophoretic deposition method to drive surface-functionalized Ag NPs into the readily-prepared mesoporous TiO_2_ layer [130]. Otherwise, plasmonic structures can be decorated onto another nanostructured semiconductor, and the hybrid material can be cast on top or beneath the bare mesoporous layer, serving as an additional light harvesting or scattering layer [131,132,133,134]. In this way, the electron screening function of the original TiO_2_ layer can be retained while taking advantage of the plasmonic layer. Moreover, Fang *et al.* have reported a facile method to fabricate mono- and bi-metallic X@TiO_2_ (X = Au, Pd, Pt) core-shell hybrid nanostructures, which can be used to replace the nanocrystalline TiO_2_, such as P25, to serve as the mesoporous layer recipe [135]. The resultant cells exhibited a 13.3% increase in the PCE and a 75% decrease in the scattering layer thickness [135]. A similar replacement method was also employed in other studies [136,137,138], in which the mesoporous layer consisted of networks of Ag-decorated porous TiO_2_ nanofibers, Ag NP-decorated N,S-co-doped TiO_2_ and Ag NP-decorated TiO_2_ spheres. The pre-synthesis of the plasmonic structure coupled mesoporous precursor allows better structural engineering and interfacial contact, as well as the tuning of the semiconductor, which enables more possibilities of further improvements.

As for the engineering of the plasmonic structures, the main considerations fall into these aspects: to improve the plasmonic profile, to increase the surface area, to facilitate the charge transport and to enhance the stability. Silver and gold are still the most chosen metals. Despite the size and shape effect, they exhibit different plasmonic profiles. Kim *et al.* fabricated a double-tiered mesoporous layer in which Ag and Au NPs were loaded in different tiers to match the different absorption peaks of the N719 dye [128]. The results demonstrated superior performance of the double-tiered structure over the single-layered counterpart, which suggested the advantages of the simultaneous employment of different kinds of metals [128]. Performance enhancement of bi-metallic structures was also reported by Dong *et al.* [118] and Yun *et al.* [119]: the incorporation of Ag-encapsulated Au nanorods and Au@Ag core/shell nanoparticles in the mesoporous film have enhanced the devices’ PCE by 5.91% → 8.43% and 7.8% → 9.7%, respectively.

The long-term photo and chemical stability is another concern for the plasmonic structures. Bare Au and Ag are vulnerable to decomposition under exposure of light and the redox electrolyte. Besides Au and Ag, other plasmonic metals, such as Al and Cu, possess even poorer resistance to harsh conditions [124,139]. Coating of protective agents over the surface is a viable means. In addition to the stability, the protective agents have to possess suitable charge transfer properties, in order to maintain or even promote the overall photochemical reactions. TiO_2_ and SiO_2_ shells are often applied and have been proven effective in promoting the long-term stability [102,120,122,123,127]. However, TiO_2_ and SiO_2_ are semiconductors that have high electric resistance, which limits the ideal device performance at some level. Carbon or conductive organic molecules are also good choices as the capping agent [121,130,140]. Polydopamine (PDA) has been recently introduced as an effective protecting agent [139]. In an Al nanodot array-based design, the incorporation of PDA nanolayers allowed for the reliable employment of LSPR of metallic Al in a highly corrosive photocatalytic redox solution. Moreover, the PDA layer provided nanoscale arrangement of organic photosensitizers over the Al surfaces. The assembly exhibited plasmon-enhanced light absorption, which resulted in a 300% efficiency increase in photochemical conversion.

Other than these coat-and-deposit strategies, the growth of plasmonic particles, deposition onto substrate and coating of protective layers can be realized simultaneously in a one-pot *in situ* reaction [141]. Ag_2_S-coated Ag NPs were deposited onto anatase TiO_2_ nanotube arrays *in situ* through the evolution of the Ag^+^-(thioglycolic acid) complex [141]. The Ag cores were in direct contact with the TiO_2_, while the exposed surface of the Ag NPs was covered by Ag_2_S. The direct contact between Ag and TiO_2_ allowed efficient interfacial charge transfer. Additionally, the hybrid nanostructure showed reasonable stability towards a high concentration poly-sulfide environment due to the protection of the Ag_2_S layer. Moreover, Ag_2_S is a small bandgap semiconductor itself, which further extended the absorption spectrum to the near-infrared region.

As for the roles played by the plasmonic structures, the three functions mentioned above could all apply. First of all, enhanced light absorption of the device was observed in almost every reference mentioned in this section. It could be direct absorption or scattering: in either way, the incident photons are more efficiently confined within the device, thus boosting the device’s PCE. Secondly, charge injection between the dye molecules and plasmonic particles, as well as between the plasmonic particles and the semiconductor substrates were evidenced in a number of reports [21,135,140,141]. In such a regard, the plasmonic particles serve as co-sensitizers and work in similar mechanisms with the organic dye. Thirdly, the strong electromagnetic field upon LSPR excitation can promote the charge carrier generation and separation of the neighboring matters, which reduces the energy loss from recombination [2,118,122,123,125,131]. Finally, it is noteworthy that the incorporation of plasmonic structures often results in open-circuit voltage increase [118,120,121,122,124,125,127,131,135,137,138,142]. This was attributed to the charging effect of the plasmonic metals, which shifted the Fermi level of the photoanode to a higher level relative to the redox potential of the electrolyte [7].

### 4.5. Perovskite Solar Cell

For the past several years, solar cells based on metal-halide perovskite absorbers, in particular organic-inorganic hybrid compounds, have been a hotspot in the photovoltaics research area. This class of solar cell has received worldwide research interest for the excellent material properties and great potential to dominate the next generation of photovoltaics market. Since the significant improvements in 2012, the power conversion efficiency of perovskite solar cells has risen from about 3% to over 20% [143]. Along with pursuing high efficiency, efforts have also been made for other attractive properties, such as environmental friendliness, flexibility, transparency and color [144]. Even though the perovskite absorbers can already absorb sunlight effectively in well-operating solar cells, there is room for optimization at long wavelengths (λ ϵ [650, 800] nm) [144,145].

**Figure 7:**
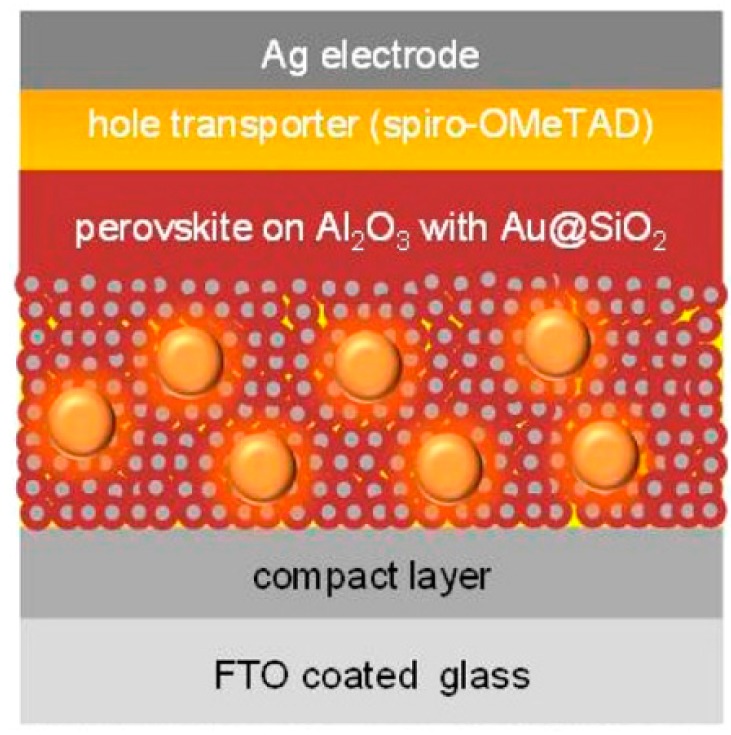
Au@SiO_2_-incorporated perovskite solar cell. Reproduced with permission [145]. Copyright 2013, American Chemical Society.

With the considerations of making use of the plasmonic enhancement, Snaith and co-workers managed to improve the PCE of the cells from an average PCE of 8.4% to 9.5%, by incorporating core–shell Au@SiO_2_ NPs into the organometal halide perovskite layer [145]. The illustration of the device structure is shown in Figure 7. Based on a series of tests and analyses, the authors attributed the improvement in PCE to the reduced exciton binding energy due to the LSPR effect, where charge generation and separation were promoted [145]. Interestingly, the authors claimed that light absorption enhancement was not taking effect in this case, since no evidence of distinguished light absorption spectra or featured plasmonic peak was observed [145]. In a later work, Lu *et al*. speculated that the narrow LSP resonant peak resulting from the Au@SiO_2_ NPs overlapped with the strong light absorption of perovskite and reported the introduction of irregular Au-Ag alloy “popcorn-shaped” NPs into perovskite solar cells [146]. The light absorption enhancement and electron transfer improvement were both evidenced. The broadband optical absorption enhancement was obtained, especially around the band edge between 720 and 820 nm. The steady-state and transient PL studies also suggested that faster charge transfer at the TiO_2_/perovskite interface was induced by the plasmonic popcorn NPs. As a result, the maximum PCE of perovskite solar cells was enhanced from 8.9% to 10.3%. Another work carried out by Hsu *et al.* employed the light scattering effect by embedding Ag nanoplates in the PEDOT:PSS layer and achieved increase in PCE from 8.5% to 9.6% [115]. Míguez *et al* conducted numerical simulation analysis of solar absorption enhancement in organic-inorganic halide perovskite films with embedded plasmonic gold nanoparticles [144]. It was derived that the relative contribution of scattering and near-field localization would vary significantly depending on the thickness of the film considered [144]. Moreover, the outcome depended on the particle size and concentration [144]. The absorption could be maximized when plasmonic near-field and light scattering effects are adequately balanced under the optimal combination of the parameters [144].

It is worth mentioning that although the perovskite solar cell has attracted vast research interest for years and there are hundreds of relevant publications by now, the number of works done on plasmonic enhancement for the perovskite solar cell is limited, *i.e.*, less than 10 to our best knowledge. There is a high potential for further development in this challenging research area. According to the literature [145], Au NPs were capped with SiO_2_ shells before incorporating into the mesoporous layer. SiO_2_ nanoparticles by themselves did not enhance the photocurrent, but rather reduced it. However, the insulating layer was essential not only for better structural and thermal stability of the nanoparticles, but also, more importantly, for preventing direct contact between Au and the hole conductor spiro-OMeTAD or the perovskite, to inhibit undesired charge movement within the device [145]. Moreover, considering that the current perovskite compounds are sensitive to the surrounding conditions, the plasmonic near-field effects, such as localized heating, may harm the durability of the functional compounds. Further improvement is needed for the perovskite material to maximize the benefit of the LSPR effect.

## 5. Conclusions

We have reviewed the recent development of plasmon-enhanced photocatalysis and photovoltaics, especially on the three major functions of the plasmonic effect, namely, light absorption, hot electron injection and near field enhancement. The plasmonic mechanisms and their photonic effects reveal that plasmonics is a very promising strategy to improve photocatalysis and photovoltaics. Significant progress has been made to develop the technology for practical application. In the future, continual research should address the following concerns:

1. Efficiency is still below satisfaction. Although plasmonic enhancement has been proven useful for PC and PV applications, effort is needed to develop the material and architecture for optimization.
2. Photo-stability and chemical-stability of plasmonic materials are poor, especially when complicated morphologies with high structural energies are required. Although many studies have reported improved durability of the plasmonic materials, the tradeoff between protection and efficiency needs to be addressed.
3. Au and Ag are effective, but expensive. Low-cost plasmonic metals, such as Al and Cu, should be investigated.
4. Our limited understanding in the interaction between plasmonic materials and electrolyte in plasmonic photovoltaics hinders the development of novel, effective materials.
5. Plasmonics is a promising approach to enhance perovskite solar cells. Further effort should be made to investigate the plasmonic materials.

Clearly, solar plasmonic PV and PC are exciting technologies and continual R & D efforts will be highly fruitful.
